# On Rényi Permutation Entropy

**DOI:** 10.3390/e24010037

**Published:** 2021-12-24

**Authors:** Tim Gutjahr, Karsten Keller

**Affiliations:** Institute of Mathematics, University of Lübeck, D-23562 Lübeck, Germany; keller@math.uni-luebeck.de

**Keywords:** Rényi entropy, permutation entropy, Kolmogorov–Sinai entropy

## Abstract

Among various modifications of the permutation entropy defined as the Shannon entropy of the ordinal pattern distribution underlying a system, a variant based on Rényi entropies was considered in a few papers. This paper discusses the relatively new concept of Rényi permutation entropies in dependence of non-negative real number *q* parameterizing the family of Rényi entropies and providing the Shannon entropy for q=1. Its relationship to Kolmogorov–Sinai entropy and, for q=2, to the recently introduced symbolic correlation integral are touched.

## 1. Introduction

### 1.1. Paper Background and Motivation

Since Bandt and Pompe [1] introduced the concept of permutation entropy (PE), it has been applied in different fields from biomedicine to econophysics (e.g., Zanin et al. [2], and Amigò et al. [3]) and developed into various directions. One relatively new variant of permutation entropy is based on Rényi entropies instead of the originally used Shannon entropy and is called Rényi permutation entropy (RPE). Roughly speaking, RPE quantifies the complexity of the distribution of ordinal patterns of some length *n* underlying a dynamical system, where ordinal patterns describe the up and down in the dynamics. As Rényi entropies depend on a parameter q∈[0,∞], there are also different choices of RPE depending on *q*.

The central aim of the paper is to discuss the asymptotics of RPE for increasing pattern length. This is motivated by the striking fact that, under certain assumptions, asymptotic PE is equal to Kolmogorov–Sinai entropy, which was first observed by Bandt et al. [4]. This paper shows that the situation for q≠1 is more complicated than that for q=1.

The paper is organized as follows. It first follows a short overview of first applications of the RPE. Section 2 provides the main definitions. The concepts of RPE are introduced in empirical and model-based settings. Moreover, RPE is discussed for some special *q*, including q=∞ as a limit case. Section 3 is devoted to the asymptotics of RPE and PE. With Corollary 1, the section contains the main new result of the paper relating RPE to Kolmogorov–Sinai entropy for q∈[0,1] and measures with maximal entropy. Its proof and a class of discriminating examples for q>1 (see Example A1) are given in Appendix A.

### 1.2. First Applications of RPE

To our best knowledge, the concept of RPE was first considered in the literature in 2015. In a study of monitoring the depth of anaesthesia by EEG, Liang et al. [5] systematically compared 12 entropy measures, with RPE among them. They reported that RPE had the best performance in distinguishing different anaesthesia states. Mammone et al. [6] discussed RPE in the context of absence epilepsy EEG. Their results suggested improved abilities in classifying ictal and interictal EEG by using RPE (with suitable parameters) instead of PE. Zunino et al. [7] introduced permutation min entropy, which is the limit of Rényi entropy for defining parameter *q* approaching to *∞* as a tool for finding temporal correlations in a time series.

Moreover, Rivero et al. [8] combined an enhanced Bayesian approach and RPE for predicting long-term time series. Following the results of Liang et al., Park et al. [9] used RPE for comparing anaesthetics given during a cesarean section with similar results as those for other entropy measures. Different variants of RPE, from weighting to multiscaling, have been applied to complex stock-market data (Zhou and Shang [10], and Chen et al. [11]). Some remarks on RPE can also be found in [12].

## 2. Rényi Entropies

### 2.1. General Entropy Concept

Given a finite index set *I* consisting of *n* elements, ${\left(p_{i}\right)}_{i\in I}\in R^{n}$ is called a *stochastic vector* if $p_{i}\geq 0$ for all i∈I and $\sum_{i\in I}p_{i}=1$. The Rènyi entropy $\mathrm{RE}({\left(p_{i}\right)}_{i\in I},q)$ of a stochastic vector ${\left(p_{i}\right)}_{i\in I}$ for q∈[0,∞] is defined by

$$\begin{array}{c} \mathrm{RE}\left({\left(p_{i}\right)}_{i\in I},q\right)=\;\left\{\begin{array}{cc} -\sum_{i\in I}p_{i}\log p_{i} & \mathrm{for}\;q=1\;\\ \frac{1}{1-q}\log\sum_{i\in I}p_{i}^{q} & \mathrm{else} \end{array}\right.. \end{array}$$

The Rényi entropy of a fixed stochastic vector monotonically decreases and is continuous with respect to *q*. It generalizes the *Shannon entropy* given in the standard case q=1. The larger that *q* is, the more the role of the largest entries in the stochastic vector is emphasized, and the smaller that *q* is, the more equal the role of all positive entries in the entropy formula is.

On the basis of the concept of Rényi entropies, we want to give precise definitions of RPE regarding both the empirical and the modelling viewpoint.

### 2.2. Empirical RPE

For n∈N, we denote the set of permutations of {0,1,…,n−1} by $S_{n}$. A vector $\left(v_{0},v_{1},\ldots ,v_{n-1}\right)\in R^{n}$ has *ordinal pattern* $\pi =\left(\pi_{0},\pi_{1},\ldots ,\pi_{n-1}\right)\in S_{n}$ if

$$\begin{array}{c} v_{\pi_{0}}\geq v_{\pi_{1}}\ldots \geq v_{\pi_{n-2}}\geq v_{\pi_{n-1}} \end{array}$$

and

$$\begin{array}{c} r_{l-1}>r_{l}\;if\;v_{r_{l-1}}=v_{r_{l}}. \end{array}$$

The latter requirement realises the uniqueness of ordinal patterns.

**Definition** **1.** *The empirical Rényi permutation entropy for q∈[0,∞] and n∈N of a time series ${\left(x_{t}\right)}_{t=0}^{N-1}$ is defined by*

$$\begin{array}{ccc} \mathrm{ePE}\left({\left(x_{t}\right)}_{t=0}^{N-1},q,n\right) & = & \mathrm{RE}({\left(p_{\pi }\right)}_{\pi \in S_{n}},q)\\ \; & = & \left\{\begin{array}{cc} -\sum_{\pi \in S_{n}}p_{\pi }\log p_{\pi } & \mathrm{for}\;q=1\;\\ \frac{1}{1-q}\log\left(\sum_{\pi \in S_{n}}p_{\pi }^{q}\right) & \mathrm{else} \end{array}\right. \end{array}$$

*with*

$$\begin{array}{ccc} p_{\pi }^{\tau } & = & \frac{\#\{t\in \left\{0,1,\ldots ,N-n\right\}|\left(x_{t},x_{t+1},\ldots ,x_{t+n-1}\right)\;\mathrm{has}\;\mathrm{ordinal}\;\mathrm{pattern}\;\pi \}}{N-n+1} \end{array}$$

*being the relative frequency of ordinal patterns π in the time series, and 0log0 and $0^{0}$ being defined by* 0.

### 2.3. RPE

On the model side, we consider a *measure-preserving dynamical system* (Ω,A,μ,T), defined as a probability space (Ω,A,μ) being equipped with a A-A-measurable map T:Ω→Ω which satisfies $\mu (T^{-1}\left(A\right))=\mu \left(A\right)$ for all A∈A. *T* and the system (Ω,A,μ,T) are called ergodic if $T^{-1}\left(A\right)=A$ for A∈A implies μ(A)∈{0,1}.

Generally, the dynamics of *T* can be related to ordinal patterns via a real-valued random variable *X* on Ω by assigning ω∈Ω the ordinal pattern of $(X\left(\omega \right),X\left(T\left(\omega \right)\right),\ldots ,X\left(T^{n-1}\left(\omega \right)\right)$. Here, *X* is interpreted as observable modelling of a measuring process. If *X* (or, more generally, a collection of random variables) has certain separation properties, the ordinal patterns obtained via *X* (or all random variables) contain much information on the given system. In the following, however, we usually assume that Ω is a subset of R, and the ordinal pattern Π(x) assigned to x∈Ω is that taken from $\left(x,T\left(x\right),\ldots ,T^{n}\left(x\right)\right)$ (this is equivalent to considering an *X* being the identity map).

For a permutation $\pi =\left(\pi_{0},\pi_{1},\cdots ,\pi_{n-1}\right)\in S_{n}$, we denote the sets of all points *x* with ordinal pattern Π(x)=π by $P_{\pi }$. From these sets, we obtain the partition

$$O\;P^{}\left(n\right)=\left\{P_{\pi }\neq Ø|\pi \in S_{n}\right\}$$

of Ω, with this being central to considering RPE on the model side. Analogously to empirical permutation entropy, we define Rényi permutation entropy for q∈[0,∞] and n∈N on the basis of $O\;P^{}\left(n\right)$ by

$$\begin{array}{ccc} \mathrm{PE}\left(T,q,n\right) & = & \mathrm{RE}({\left(\mu \left(P_{\pi }\right)\right)}_{\pi \in S_{n}},q)\\ \; & = & \left\{\begin{array}{cc} -\sum_{\pi \in S_{n}}\mu \left(P_{\pi }\right)\log\mu \left(P_{\pi }\right) & \mathrm{for}\;q=1\;\\ \frac{1}{1-q}\log\left(\sum_{\pi \in S_{n}}\mu {\left(P_{\pi }\right)}^{q}\right) & \mathrm{else}. \end{array}\right. \end{array}$$

### 2.4. Estimation

Given an orbit

$$\left(x_{0},x_{1},\ldots ,x_{N-2},x_{N-1}\right)=\left(x,T\left(x\right),\ldots ,T^{N-2}\left(x\right),T^{N-1}\left(x\right)\right)$$

of some x∈Ω, it is natural to estimate $\mu (P_{\pi })$ for $\pi \in S_{n}$ and $\mathrm{PE}\left(T,q,n\right)$ by $p_{\pi }$ and $\mathrm{ePE}\left({\left(x_{t}\right)}_{t=0}^{N-1},q,n\right)$, respectively. In the case that *T* is ergodic, by Birkhoff’s ergodic theorem, the corresponding estimators are asymptotically consistent. This particularly means that

$$\lim_{N\to \infty }\mathrm{ePE}\left({\left(T^{t}\left(x\right)\right)}_{t=0}^{N-1},q,n\right)\;=\mathrm{PE}\left(T,q,n\right)$$

for μ-almost all x∈Ω.

### 2.5. RPE for Special Parameters q

In the following, we discuss the RPE for some special parameters *q*, and touch the general concept of Rényi entropies.

q=0: Rényi entropy for q=0 is the well-known *Hartley entropy*, and the RPE of a measure preserving dynamical system is no more than the logarithm of the number of ordinal patterns appearing with positive probability. Rényi entropy for q=0 is also called *max entropy* since it is maximal among Rényi entropies.
q=1: This case providing the standard (Shannon) permutation entropy has been discussed in various papers both from a theoretical and an application viewpoint. We particularly refer to the literature mentioned in several parts of this paper.
q=2: Rényi entropy for q=2, also called *quadratic entropy* or *collision entropy*, is used in different fields. It is obviously related to the *Simpson index*$\sum_{i=1}^{n}p_{i}^{2}$ given for a stochastic vector ${\left(p_{i}\right)}_{i=1}^{n}$ and used as a diversity measure in ecology (see [13]). Given a measure-preserving dynamical system (Ω,A,μ,T), we look at the RPE for q=2.

By Fubini’s theorem, it holds that

$$\begin{array}{ccc} \mathrm{PE}\left(T,2,n\right) & = & -\log\left(\sum_{\pi \in S_{n}}\mu {\left(P_{\pi }\right)}^{2}\right)\\ \; & = & -\log\left(\sum_{\pi \in S_{n}}{\left(\int 1_{P_{\pi }}\;d\mu \right)}^{2}\right)\\ \; & = & -\log\left(\sum_{\pi \in S_{n}}\int \int 1_{P_{\pi }}\left(\omega_{1}\right)\;1_{P_{\pi }}\left(\omega_{2}\right)\;d\mu \left(\omega_{1}\right)d\mu \left(\omega_{2}\right)\right)\\ \; & = & -\log\int \int \sum_{\pi \in S_{n}}\left(1_{P_{\pi }}\left(\omega_{1}\right)\;1_{P_{\pi }}\left(\omega_{2}\right)\right)\;d\mu \left(\omega_{1}\right)d\mu \left(\omega_{2}\right)\\ \; & = & -\log\int \sum_{\pi \in S_{n}}\left(1_{P_{\pi }}\left(\omega_{1}\right)\;1_{P_{\pi }}\left(\omega_{2}\right)\right)\;d\mu^{2}\left(\omega_{1},\omega_{2}\right)\\ \; & = & -\log S^{n} \end{array}$$

with

$$S^{n}=\mu^{2}\left\{\left(\omega_{1},\omega_{2}\right)\in \Omega \times \Omega |\Pi \left(\omega_{1}\right)=\Pi \left(\omega_{2}\right)\right\}.$$

Here, $1_{A}$ stands for the indicator of a set *A* assigning a point the value 1 if it belongs to *A* and value 0 otherwise, and $\mu^{2}$ denotes the product measure of μ with itself.

So, $\mathrm{PE}\left(T,2,n\right)$ is related to the probability that the ordinal patterns of length *n* of two independently (with respect to μ) chosen points coincide.

A natural estimation of $S^{n}$ based on a finite orbit

$$\left(x_{0},x_{1},\ldots ,x_{N-2},x_{N-1}\right)=\left(x,T\left(x\right),\ldots ,T^{N-2}\left(x\right),T^{N-1}\left(x\right)\right)$$

of some x∈Ω is given by

(1) $$\frac{2}{\left(N+1-n)(N-n\right)}\#\left\{\left(s,t\right)|0\leq s<t\leq N-1,\;\Pi \left(x_{s}\right)=\Pi \left(x_{t}\right)\right\}$$

providing the relative frequency of pairs in the orbit with coinciding (completely defined) ordinal patterns. This qualifies the RPE for q=2 as a recurrence measure.

Quantity (1) was introduced by Caballero et al. [14] as the *symbolic correlation integral* in the context of a stochastic process and studied mainly in the i.i.d. case.

q=∞: It is well-known that the Rényi entropy of a stochastic vector ${\left(p_{i}\right)}_{i\in I}$ for q→∞ converges to value
  (2) $$\mathrm{RE}\left({\left(p_{i}\right)}_{i\in I},\infty \right):=\lim_{q\to \infty }\mathrm{RE}\left({\left(p_{i}\right)}_{i\in I},q\right)=-\log\max_{i\in I}p_{i}.$$
  This fact can be used to reconstruct a stochastic vector up to permuting its components from its Rényi entropies for an unbounded sequence ${\left(q_{n}\right)}_{n\in N}$ (see Appendix A). Since $\mathrm{RE}\left({\left(p_{i}\right)}_{i\in I},\infty \right)\leq \mathrm{RE}\left({\left(p_{i}\right)}_{i\in I},q\right)$ for all q∈[0,∞], number $\mathrm{RE}({\left(p_{i}\right)}_{i\in I},\infty )$ is called *min entropy* of ${\left(p_{i}\right)}_{i\in I}$. Applications of min entropy in the permutation entropy context can be found in Zunino et al. [7]. In the following, we further assume that q<∞.

## 3. Asymptotics of RPE and PE

As already mentioned, there is a strong relationship between Kolmogorov–Sinai entropy and PE. The result of Takens and Verbitskiy [15] that, for q>1, Kolmogorov–Sinai entropy can be expressed by a limit on the basis of Rényi entropies instead of Shannon entropies suggests the question whether PE can be similarly replaced by RPE in that relationship. This question addresses the asymptotics of RPE, and the general nature of RPE is thus in the centre of this section.

### 3.1. Kolmogorov–Sinai Entropy via Rényi Entropies

Definitions and statements of this subsection go back to Takens and Verbitsky [15]. Many considerations of this paper are related to partitions of Ω. We generally assume that, in a context where a σ-algebra on Ω is specified, such partitions are contained in it.

Let (Ω,A,μ,T) now be a measure-preserving dynamical system, and consider a finite partition $P={\left\{P_{i}\right\}}_{i\in I}$ of Ω. For n∈N and multi-indices $i=\left(i_{0},i_{1},\ldots ,i_{n-1}\right)\in I^{n}$ define the sets

$$P\left(i\right):=\overset{n-1}{\underset{t=0}{⋂}}T^{-t}\left(P_{i_{t}}\right)=P_{i_{0}}\cap T^{-1}\left(P_{i_{1}}\right)\cap \ldots T^{-n+1}\left(P_{i_{n-1}}\right)$$

forming the partition

$$P^{\left(n\right)}=\left\{P\left(i\right)\neq Ø\;|\;i\in \left(i_{0},i_{1},\ldots i_{n-1}\right)\in I^{n}\right\}$$

of Ω. For q∈[0,∞], the *generalized entropy rate* of *T* *with respect to partition* P is defined as

(3) $$h\left(T,P,q\right):=\underset{n\to \infty }{lim inf}\frac{1}{n}H\left(P^{\left(n\right)},q\right)$$

with

$$\begin{array}{c} H\left(Q,q\right)=\left\{\begin{array}{cc} -\sum_{Q\in Q}\mu \left(Q\right)\log\mu \left(Q\right) & \mathrm{for}q=1\;\\ \frac{1}{1-q}\log\left(\sum_{Q\in Q}\mu {\left(Q\right)}^{q}\right) & \mathrm{else} \end{array}\right. \end{array}$$

for a finite partition Q of Ω. Generalized Kolmogorov–Sinai entropy for q∈[0,∞] is defined as the supremum of generalized entropy rates taken over all finite partitions:

$$h\left(T,q\right):=\underset{P\;\mathrm{finite}\;\mathrm{partition}\;\mathrm{of}\;\Omega }{\sup}h\left(T,P,q\right).$$

(Standard) Kolmogorov–Sinai entropy is given by

h(T):=h(T,1).

In the case of q=1, the limit inferior in (3) can be replaced by a limit and that, for Ω being an interval and A being the Borel σ-algebra, Kolmogorov–Sinai entropy is already determined by *finite interval partitions* defined as finite partitions consisting of intervals (e.g., [16]):

$$h\left(T\right)=\underset{P\;\mathrm{finite}\;\mathrm{interval}\;\mathrm{partition}\;\mathrm{of}\;\Omega }{\sup}h\left(T,P,1\right).$$

The following theorem of Takens and Verbitskiy [15] was originally proved for invertible systems; however, it also holds true for noninvertible systems (see Verbitskiy [17]). Assumption (i) of ergodicity can be relaxed (see Takens and Verbitskiy [18]); however, we do not go into the technical details.

**Theorem** **1.** *Let (Ω,A,μ) be a standard-probability space and T:Ω→Ω an aperiodic and ergodic measure-preserving function. Then,*

h(T,q)=h(T)

*holds true for all q∈[1,∞]. Additionally, h(T)>0 implies*

h(T,q)=∞

*for all q∈[0,1].*

Here, *T* is called *aperiodic* if the set of periodic points has measure zero with respect to μ. The property of a probability space to be standard is a relatively technical one; however, it is not very restrictive since it is principally satisfied for the most common probability spaces (e.g., Walters [16]).

### 3.2. Kolmogorov–Sinai Entropy and RPE

In order to discuss the relationship between RPE and Kolmogorov–Sinai entropy of a measure-preserving dynamical system (Ω,A,T,μ), we define *lower* and *upper* Rényi permutation entropies $\underline{\mathrm{PE}}\left(T,q\right)$ and $\bar{\mathrm{PE}}\left(T,q\right)$ for q∈[0,∞] as

$$\underline{\mathrm{PE}}\left(T,q\right)=\underset{n\to \infty }{lim inf}\frac{1}{n}H\left(O\;P^{}\left(n\right),q\right)$$

and

$$\bar{\mathrm{PE}}\left(T,q\right)=\underset{n\to \infty }{lim sup}\frac{1}{n}H\left(O\;P^{}\left(n\right),q\right),$$

respectively. We write $\underline{\mathrm{PE}}\left(T\right)$ and $\bar{\mathrm{PE}}\left(T\right)$ in the case of q=1. Both $\underline{\mathrm{PE}}\left(T,q\right)$ and $\bar{\mathrm{PE}}\left(T,q\right)$ monotonically decreasee with respect to *q* by the definition of Rényi entropies.

q=1: The celebrated result of Bandt et al. [4] that, for piecewise continuous and monotone interval maps, the permutation and Kolmogorov-Sinai entropy coincide is the motivation for the following discussion. Here, we state the more general version of the result proved in Gutjahr and Keller [19], but afterwards return to the case of piecewise monotone interval maps. In the following, we call a subset of Ω⊂R interval if it is the intersection of an interval of R with Ω or a one point set.

**Theorem** **2** ([19]). *Let (Ω,B,μ,T) be a measure-preserving dynamical system, with Ω⊆R being compact, and B being the Borel σ-algebra on *Ω*. If there exists a finite partition M or a countable partition M with H(M)<∞ of *Ω* into intervals, such that T is monotone on each of the intervals, then*

$$\bar{\mathrm{PE}}\left(T\right)=h\left(T\right).$$

Theorem 2 covers interval maps, since a noncompact Ω can be replaced by compactification without substantially changing the structure of the given system.

q>1: In light of the statement of Takens and Verbitskiy [15] mentioned above, it is a natural question whether also $\bar{\mathrm{PE}}\left(T,q\right)=h\left(T,q\right)\left(=h\left(T\right)\right)$. The general answer is no. Examples with $\bar{\mathrm{PE}}\left(T,q\right)<h\left(T,q\right)$ covering all q>1 are given by Example A1 in Appendix B.2.

q<1: We also look at case q<1 in the class of maps considered by Bandt et al. in [4]. For this, let Ω be an interval, B the Borel σ-algebra on it, and M be a finite partition of Ω into intervals on each of which *T* is monotone and continuous. For such a map, it was shown in [4] that

(4) $$\lim_{n\to \infty }\frac{1}{n}\log\#\left\{P_{\pi }\in O\;P^{}\left(n\right)|P_{\pi }\neq Ø\right\}=\lim_{n\to \infty }\frac{1}{n}\log\#\left\{M\in M^{\left(n\right)}|M\neq Ø\right\}$$

holds true. Using the fact that Rényi entropy monotonically decreases in *q*, this implies

$$\begin{array}{ccc} \bar{\mathrm{PE}}\left(T,q\right) & \leq & \bar{\mathrm{PE}}\left(T,0\right)\\ & = & \underset{n\to \infty }{lim sup}\frac{1}{n}\log\left(\sum_{P_{\pi }\in O\;P^{}\left(n\right)}\mu {\left(P_{\pi }\right)}^{0}\right)\\ \; & = & \underset{n\to \infty }{lim sup}\frac{1}{n}\log\#\left\{P_{\pi }\in O\;P^{}\left(n\right)|\mu \left(P_{\pi }\right)>0\right\}\\ \; & \leq & \lim_{n\to \infty }\frac{1}{n}\log\#\left\{P_{\pi }\in O\;P^{}\left(n\right)|P_{\pi }\neq Ø\right\}\\ \; & = & \lim_{n\to \infty }\frac{1}{n}\log\#\left\{M\in M^{\left(n\right)}|M\neq Ø\right\} \end{array}$$

for all q∈[0,∞]. Let us summarize:

**Proposition** **1.** *Let (Ω,B,μ,T) be a measure-preserving dynamical system, with Ω⊆R being an interval, and B being the Borel σ-algebra on it. Suppose that M is a finite partition of *Ω* into intervals such that T is monotone and continuous on each of the intervals. Then*

$$\bar{\mathrm{PE}}\left(T,q\right)\leq \lim_{n\to \infty }\frac{1}{n}\log\#\left\{M\in M^{\left(n\right)}|M\neq Ø\right\}$$

*holds true for all q∈[0,∞].*

Quantity ${lim sup}_{n\to \infty }\frac{1}{n}\log\#\left\{P_{\pi }\in O\;P^{}\left(n\right)|P_{\pi }\neq Ø\right\}$ could be considered to be a topological version of permutation entropy. This is justified by (4) for *T*, as defined above (4), and the following: If *T* is continuous on all of Ω, Misiurewicz and Szlenk showed that $\lim_{n\to \infty }\frac{1}{n}\log\#\left\{M\in M^{\left(n\right)}|M\neq Ø\right\}$ is equal to the topological entropy of *T* [20]. For the definition of topological entropy and the following, see, e.g., [16].

By variation principle, the topological entropy of a map *T* on a compact Hausdorff space is equal to the supremum of the Kolmogorov–Sinai entropy of all Borel measures for which *T* is measure-preserving (e.g., [16]). Often, topological entropy is assumed by the Kolmogorov–Sinai of such a measure. Generally, given a continuous map *T* on a metric space, a corresponding Borel measure being measure-preserving has maximal entropy if its Kolmogorov–Sinai entropy coincides with the topological entropy of *T*.

On the basis of the discussion above, we show the following statement (see Appendix B.1).

**Corollary** **1.** *Let (Ω,B,μ,T) be a measure-preserving dynamical system, with Ω⊆R being an interval, and B being the Borel σ-algebra on *Ω*. Suppose that T is continuous, and there exists a finite partition of *Ω* into intervals, such that T is monotone on each of those intervals, and μ is a measure of maximal entropy. Then*

$$\underline{\mathrm{PE}}\left(T,q\right)=\bar{\mathrm{PE}}\left(T,q\right)=\underset{P\;finite\;interval\;partition\;of\;\Omega }{\sup}h\left(T,P,q\right)=h\left(T\right)$$

*holds true for all q∈[0,1].*

## 4. Conclusions

In this paper, we looked more closely at the recently introduced and used Rényi variant of permutation entropy, depending on a parameter q∈[0,∞], which is called Rényi permutation entropy (RPE) here. Giving a summary of first applications of RPE, and discussing RPE for some special parameter *q*, we mainly focused on the asymptotics of RPE for ordinal pattern length going to *∞*. This was motivated by the fact that the usual permutation entropy (PE) often asymptotically coincides with Kolmogorov–Sinai entropy, and that, for q>1, Kolmogorov–Sinai entropy can be defined by Rényi entropies instead of Shannon entropies.

This paper showed that, for q>1, asymptotics of RPE can be different from that of PE, meaning that, for long ordinal patterns, the nature of RPE is also not the same as that of PE. One the other hand, it is interesting that, for continuous piecewise monotone interval maps with a measure of maximal entropy and q<1, asymptotics of RPE and PE are the same. Results indicate that the behaviour of general RPE is more specific than that of PE, although the asymptotics of PE is not completely understood. Further work for the better understanding of RPE for large pattern lengths is necessary.

The content of this paper is more or less purely mathematical, but in a certain sense, it justifies the application of RPE in dynamical systems and time series besides PE. Some of the applications mentioned at the beginning of the paper underline the benefit of using RPE. There is, however, the other interesting point that special *q* address special features; so, for example, q=2 is related to recurrence. The symbolic correlation integral related to case q=2 is a U-statistic, which is helpful in the statistical analysis of the corresponding entropy. Work on utilizing this fact for testing for asymmetry in temporal data is in progress.

## Appendix A. Reconstruction of Stochastic Vectors from Rényi Entropies

Let ${\left(p_{i}\right)}_{i=1}^{m}$ be a stochastic vector, and let ${\left(q_{n}\right)}_{n\in N}$ be an unbounded sequence of positive numbers, such that $\mathrm{RE}({\left(p_{i}\right)}_{i=1}^{m},q_{n})$ is known for all n∈N. Assume that $p_{1}\leq p_{2}\leq \ldots \leq p_{m}$. The reconstruction we give is inductive.

If $p_{1},p_{2},\ldots ,p_{r-1}$ are known for some r∈{1,2,…,m} and $\sum_{j=1}^{r-1}p_{j}<1$, then ${\left(\frac{p_{i}}{1-\sum_{j=1}^{r-1}p_{j}}\right)}_{i=r}^{m}$ is a stochastic vector and

$$\mathrm{RE}\left({\left(p_{i}\right)}_{i=1}^{m},q_{n}\right)=\frac{1}{1-q_{n}}\log\left(\sum_{i=1}^{r-1}p_{i}^{q_{n}}+\sum_{i=r}^{m}p_{i}^{q_{n}}\right)$$

for all n∈N. This implies

$$\sum_{i=r}^{m}p_{i}^{q_{n}}=e^{\left(1-q_{n}\right)\;\mathrm{RE}\left({\left(p_{i}\right)}_{i=1}^{m},\;q_{n}\right)}-\sum_{i=1}^{r-1}p_{i}^{q_{n}}$$

for all n∈N. From this and (2), we obtain

$$\begin{array}{ccc} -\log\left(\frac{p_{r}}{1-\sum_{j=1}^{r-1}p_{j}}\right) & = & \underset{n\in N}{\inf}\mathrm{RE}\left({\left(\frac{p_{i}}{1-\sum_{j=1}^{r-1}p_{j}}\right)}_{i=r}^{m},\;q_{n}\right)\\ \; & = & \underset{n\in N}{\inf}\left(\frac{1}{1-q_{n}}\log\left(\sum_{i=r}^{m}{\left(\frac{p_{i}}{1-\sum_{j=1}^{r-1}p_{j}}\right)}^{q_{n}}\right)\right)\\ \; & = & \underset{n\in N}{\inf}\left(\frac{1}{1-q_{n}}\log\sum_{i=r}^{m}p_{i}^{q_{n}}-\frac{q_{n}}{1-q_{n}}\log\left(1-\sum_{j=1}^{r-1}p_{j}\right)\right), \end{array}$$

hence

$$-\log p_{r}=\underset{n\in N}{\inf}\frac{1}{1-q_{n}}\left(\log\sum_{i=r}^{m}p_{i}^{q_{n}}-\log\left(1-\sum_{j=1}^{r-1}p_{j}\right)\right)$$

and

$$\log p_{r}=\underset{n\in N}{\sup}\frac{1}{q_{n}-1}\left(\log\left(e^{\left(1-q_{n}\right)\;\mathrm{RE}\left({\left(p_{j}\right)}_{j=1}^{m},\;q_{n}\right)}-\sum_{i=1}^{r-1}p_{i}^{q_{n}}\right)-\log\left(1-\sum_{j=1}^{r-1}p_{j}\right)\right)$$

Thus, $p_{r}$ is also known.

## Appendix B. Proofs

Some of the following statements are only given on a level of generality required here. In some cases, for more general statements and further details, we refer to Gutjahr [21].

### Appendix B.1. Proof of Corollary 1

First, if a finite partition P of Ω is finer than another one, Q, then H(P,q)≥H(Q,q) for all q∈[0,∞]. This is well-known and easily follows from the concavity and convexity of $x\mapsto x^{q}$ for q<1 and q>1, respectively, and from the convexity of x↦xlogx for q=1 on [0,∞].

Given two finite partitions P,Q of Ω, let P∨Q={P∩Q≠Ø∣P∈P,Q∈Q} be the largest common refinement of P and Q. Further, for a partition P of Ω and some Q∈A, let Δ(P|Q):={P∈P∣μ(P∩Q)>0} and #Δ(P|Q) the number of elements of Δ(P|Q).

The proof of Corollary 1 is built on the two following lemmata.

**Lemma** **A1.**
*Let (Ω,A,μ) be a probability space, and P,Q two finite partitions of *Ω*. Then*

$$H\left(P\vee Q,q\right)\leq H\left(Q,q\right)+\left\{\begin{array}{cc} \sum_{Q\in Q}\mu \left(Q\right)\cdot \log\left(\#\Delta \left(P|Q\right)\right) & \mathrm{for}\;q=1\\ \max_{Q\in Q}\log\left(\#\Delta \left(P|Q\right)\right) & \mathrm{for}\;q\in \;[0,\infty ]\backslash \{1\}. \end{array}\right.$$

**Proof.** Consider function f:[0,∞]→R with $f\left(x\right)=x^{q}$. Since *f* is concave for q<1 and convex for q>1, Jensen’s inequality implies

$$\sum_{P\in \Delta (P|Q)}\frac{f(\mu (P\cap Q))}{\#\Delta (P|Q)}\left\{\begin{array}{cc} \leq f\left(\sum_{P\in \Delta (P|Q)}\frac{\mu (P\cap Q)}{\#\Delta (P|Q)}\right) & \mathrm{if}\;q<1\\ \geq f\left(\sum_{P\in \Delta (P|Q)}\frac{\mu (P\cap Q)}{\#\Delta (P|Q)}\right) & \mathrm{if}\;q>1 \end{array}\right.$$

for all Q∈Q. Using this and the fact that function $\frac{-1}{q-1}\log$ monotonically increases for q<1 and decreases for q>1 provides

$$\begin{array}{ccc} H(P\vee Q,q) & = & \frac{-1}{q-1}\log\left(\sum_{Q\in Q}\sum_{P\in P}\mu {\left(P\cap Q\right)}^{q}\right)\\ \; & = & \frac{-1}{q-1}\log\left(\sum_{Q\in Q}\sum_{P\in \Delta (P|Q)}\mu {\left(P\cap Q\right)}^{q}\right)\\ \; & = & \frac{-1}{q-1}\log\left(\sum_{Q\in Q}\#\Delta \left(P|Q\right)\sum_{P\in \Delta (P|Q)}\frac{f(\mu (P\cap Q))}{\#\Delta (P|Q)}\right)\\ \; & \leq & \frac{-1}{q-1}\log\left(\sum_{Q\in Q}\#\Delta \left(P|Q\right)\cdot f\left(\sum_{P\in \Delta (P|Q)}\frac{\mu (P\cap Q)}{\#\Delta (P|Q)}\right)\right)\\ \; & = & \frac{-1}{q-1}\log\left(\sum_{Q\in Q}\#\Delta \left(P|Q\right)\cdot f\left(\frac{\mu (Q)}{\#\Delta (P|Q)}\right)\right)\\ \; & = & \frac{-1}{q-1}\log\left(\sum_{Q\in Q}\#\Delta {\left(P|Q\right)}^{1-q}\cdot \mu {\left(Q\right)}^{q}\right)\\ \; & \leq & \frac{-1}{q-1}\log\left({\left(\max_{Q\in Q}\#\Delta \left(P|Q\right)\right)}^{1-q}\cdot \sum_{Q\in Q}\mu {\left(Q\right)}^{q}\right)\\ \; & = & \frac{-1}{q-1}\log\left({\left(\max_{Q\in Q}\#\Delta \left(P|Q\right)\right)}^{1-q}\right)+\frac{-1}{q-1}\log\left(\sum_{Q\in Q}\mu {\left(Q\right)}^{q}\right)\\ \; & = & \max_{Q\in Q}\log\left(\#\Delta \left(P|Q\right)\right)+H\left(Q,q\right) \end{array}$$

for all q≠1. For q=1, consider function g:[0,∞]→[0,∞] with g(x)=xlog(x), which is convex. Therefore, by Jensen’s inequality, it follows that

$$\begin{array}{cc} \; & \sum_{P\in \Delta (P|Q)}\mu \left(P\cap Q\right)\log\left(\mu \left(P\cap Q\right)\right)\\ = & \#\Delta \left(P|Q\right)\sum_{P\in \Delta (P|Q)}\frac{1}{\#\Delta (P|Q)}\cdot g\left(\mu \left(P\cap Q\right)\right)\\ \geq & \#\Delta \left(P|Q\right)\cdot g\left(\sum_{P\in \Delta (P|Q)}\frac{1}{\#\Delta (P|Q)}\cdot \mu \left(P\cap Q\right)\right)\\ = & \#\Delta \left(P|Q\right)\cdot g\left(\frac{\mu (Q)}{\#\Delta (P|Q)}\right)\\ = & \#\Delta \left(P|Q\right)\cdot \frac{\mu (Q)}{\#\Delta (P|Q)}\cdot \log\left(\frac{\mu (Q)}{\#\Delta (P|Q)}\right)\\ = & \mu \left(Q\right)\cdot \left(\log(\mu (Q))-\log(\#\Delta (P|Q))\right). \end{array}$$

Using the above inequality provides

$$\begin{array}{ccc} H(P\vee Q) & = & -\sum_{Q\in Q}\sum_{P\in P}\mu \left(P\cap Q\right)\log\left(\mu \left(P\cap Q\right)\right)\\ \; & = & -\sum_{Q\in Q}\sum_{P\in \cdot (P|Q)}\mu \left(P\cap Q\right)\log\left(\mu \left(P\cap Q\right)\right)\\ \; & \leq & -\sum_{Q\in Q}\mu \left(Q\right)\cdot \left(\log(\mu (Q))-\log(\#\Delta (P|Q))\right)\\ \; & = & H\left(Q\right)+\sum_{Q\in Q}\mu \left(Q\right)\cdot \log\left(\#\Delta \left(P|Q\right)\right). \end{array}$$

 □

The following lemma was proved in Gutjahr and Keller [19] (see Lemma A1). We present it here in a slightly weakened form since we do not need the full generality.

**Lemma** **A2.**
*Let (Ω,B,μ,T) be a measure-preserving dynamical system, with Ω⊆R being an interval and B being the Borel σ-algebra on *Ω*. Further, let P be a finite interval partition of *Ω*. Then for all $P_{\pi }\in O\;P^{}\left(n\right)$*

#Δ(P(n)|Pπ)≤ n+#P−1#P−1

*holds true.*

We can now finalise the proof of Corollary 1. Given the assumptions of the corollary, by Proposition 1 and the monotony of the Rényi entropy with respect to *q*, we have

$$\begin{array}{ccc} \underline{\mathrm{PE}}\left(T,q\right) & \leq & \bar{\mathrm{PE}}\left(T,q\right)\\ & \leq & \lim_{n\to \infty }\frac{1}{n}\log\#\left\{M\in M^{\left(n\right)}|M\neq Ø\right\}\\ & = & h(T)\\ & = & \underset{P\;\mathrm{finite}\;\mathrm{interval}\;\mathrm{partition}\;\mathrm{of}\;\Omega }{\sup}h\left(T,P\right)\\ & \leq & \underset{P\;\mathrm{finite}\;\mathrm{interval}\;\mathrm{partition}\;\mathrm{of}\;\Omega }{\sup}h\left(T,P,q\right) \end{array}$$

for all q∈[0,1]. So, it remains to show that

$$\underline{\mathrm{PE}}\left(T,q\right)\geq h\left(T,P,q\right)$$

is true for all q∈[0,1] and for all finite interval partitions P of Ω.

Fixing some q∈[0,1] and some finite interval partition of Ω, Lemmas A1 and A2 yield

H(P(n),q)≤H(P(n)∨OP(n),q)≤H(OP(n),q)+maxπ∈Snlog(#Δ(P(n)|Pπ))≤H(OP(n),q)+maxπ∈Snlogn+#P−1#P−1≤H(OP(n),q)+maxπ∈Snlog(n+#P−1)#P−1≤H(OP(n),q)+(#P−1)log(n+#P−1)

for all q∈[0,1]. Hence,

$$\begin{array}{ccc} \underline{\mathrm{PE}}\left(T,q\right) & = & \underset{n\to \infty }{lim inf}\frac{1}{n}H\left(O\;P^{}\left(n\right),q\right)\\ \; & \geq & \underset{n\to \infty }{lim inf}\frac{1}{n}\left[H\left(P^{\left(n\right)},q\right)-\left(\#P-1\right)\log\left(n+\#P-1\right)\right]\\ \; & = & \underset{n\to \infty }{lim inf}\frac{1}{n}H\left(P^{\left(n\right)},q\right)\;=h\left(T,P,q\right). \end{array}$$

### Appendix B.2. Counter Examples

In order to give the examples after Theorem 2, we start with two lemmata.

**Lemma** **A3.**
*Let (Ω,B,μ,T) be a measure-preserving dynamical system with Ω⊆R and B being the Borel σ-algebra on *Ω*. Further, let $P={\left\{P_{i}\right\}}_{i\in I}$ be a finite partition of Ω. Then, for all q∈[1,∞],*

$$\bar{\mathrm{PE}}\left(T,q\right)\leq h\left(T,P,q\right)+\underset{n\to \infty }{lim sup}\frac{1}{n}\max_{i\in I^{n}}\log\left(\#\Delta \left(O\;P^{}\left(n\right)|P\left(i\right)\right)\right)$$

*hold true.*

**Proof.** Let $P={\left\{P_{i}\right\}}_{i\in I}$ be a finite partition of Ω. Then, Lemma A1 provides

$$H\left(O\;P^{}\left(n\right),q\right)\leq H\left(O\;P^{}\left(n\right)\vee P^{\left(n\right)},q\right)\leq H\left(P^{\left(n\right)},q\right)+\max_{i\in I^{n}}\log\left(\#\Delta \left(O\;P^{}\left(n\right)|P\left(i\right)\right)\right)$$

for all q>1 and n∈N. Dividing both sides by *n* and taking the limit superior n→∞ finishes the proof.   □

The following lemma was shown in [22] (see Lemma 2.2.).

**Lemma** **A4.**
*Let T:Ω→Ω be a map on Ω⊆R. Assume that $M={\left\{M_{i}\right\}}_{i\in I}$ is a finite partition of *Ω* into sets on each of which T is monotone. Then, for all n∈N and multi-indices $i=\left(i_{0},i_{1},\ldots ,i_{n-1}\right)\in I^{n}$*

$$\#\Delta \left(O\;P^{}\left(n\right)|M\left(i\right)\right)\leq 2^{\#\{s\in \left\{0,1,\cdots ,n-2\right\}|i_{s}=i_{n-1}\}}$$

*holds true.*

As a consequence of the two lemmata, we obtain an upper bound of RPE that is important for the following.

**Corollary** **A1.**
*Let (Ω,B,μ,T) be a measure-preserving dynamical system with Ω⊆R. Assume that M is a finite partition of *Ω* into sets on each of which T is monotone. Then, for all q∈[0,∞]*

$$\bar{\mathrm{PE}}\left(T,q\right)\leq h\left(T,M,q\right)+\log2.$$

*holds true.*

**Proof.** We have

$$\bar{\mathrm{PE}}\left(T,q\right)\leq h\left(T,M,q\right)+\underset{n\to \infty }{lim sup}\frac{1}{n}\max_{i\in I^{n}}\log\left(\#\Delta \left(O\;P^{}\left(n\right)|M\left(i\right)\right)\right)$$

according to Lemma A3. Lemma A4 provides

$$\#\Delta \left(O\;P^{}\left(n\right)|M\left(i\right)\right)\leq 2^{\#\{s\in \left\{0,1,\cdots ,n-2\right\}|i_{s}=i_{n-1}\}}\leq 2^{n-1}$$

for all n∈N and $i\in I^{n}$. Combining the above statements yields

$$\begin{array}{ccc} \bar{\mathrm{PE}}\left(T,q\right) & = & h\left(T,M,q\right)+\underset{n\to \infty }{lim sup}\left(n-1\right)/n\log2\\ & = & h(T,M,q)+\log2. \end{array}$$

 □

We use the following fact about the Rényi entropies of stochastic vectors:

**Lemma** **A5.**
*For all q∈[1,∞] and all c>0, there exists a stochastic vector p with*

H(p)−H(p,q)≥c.

**Proof.** Let c>0 and q>1. Choose N∈N, such that

$$N\geq 2\cdot \left(\frac{c}{\log(2)}+\frac{q}{q-1}\right)$$

holds true. Consider stochastic vector $p=\left(p_{1},p_{2},\ldots ,p_{2^{N}+1}\right)\in {\left[0,1\right]}^{2^{N}+1}$ with

$$p_{1}=\frac{1}{2}$$

and

$$p_{i}=\frac{1}{2^{N+1}}\;\mathrm{for}\;i\in \left\{2,3,\ldots ,2^{N}+1\right\}.$$

Then,

$$\begin{array}{ccc} H(p) & = & -\sum_{i=1}^{2^{N}+1}p_{i}\log\left(p_{i}\right)\\ & = & -2^{N}\cdot \frac{1}{2^{N+1}}\log\left(\frac{1}{2^{N+1}}\right)-\frac{1}{2}\log\left(\frac{1}{2}\right)\\ & = & \frac{N+1}{2}\log\left(2\right)+\frac{1}{2}\log\left(2\right)\\ & \geq & \frac{N}{2}\log\left(2\right) \end{array}$$

and

$$\begin{array}{ccc} H(p,q) & = & \frac{-1}{q-1}\log\left(\sum_{i=1}^{2^{N}+1}p_{i}^{q}\right)\\ & = & \frac{-1}{q-1}\log\left(2^{N}\cdot \frac{1}{2^{(N+1)q}}+\frac{1}{2^{q}}\right)\\ & \leq & \frac{-1}{q-1}\log\left(\frac{1}{2^{q}}\right)\\ & = & \frac{q}{q-1}\log\left(2\right). \end{array}$$

Hence,

$$H\left(p\right)-H\left(p,q\right)\geq \log\left(2\right)\cdot \left(\frac{N}{2}-\frac{q}{q-1}\right)\geq c.$$

 □

Recall that a (one-sided) Bernoulli shift σ is a map defined on ${\left\{0,1,\ldots ,N-1\right\}}^{\infty }$ for some N∈N by

$$\sigma \left({\left(b_{n}\right)}_{n=1}^{\infty }\right)={\left(b_{n}\right)}_{n=2}^{\infty }$$

for ${\left(b_{n}\right)}_{n=1}^{\infty }\in {\left\{0,1,\ldots ,N-1\right\}}^{\infty }$. Given a stochastic vector $p=\left(p_{0},p_{1},\ldots ,p_{N-1}\right)\in {\left[0,1\right]}^{N}$, one considers the product measure $\mu_{p}$ associated to p. More precisely, $\mu_{p}$ is the probability measure on the smallest σ-algebra containing all cylinder sets

$$C_{k}\left(a_{1},a_{2},\ldots ,a_{k}\right)=\left\{{\left(b_{n}\right)}_{n=1}^{\infty }\in {\left\{0,1,\ldots ,N-1\right\}}^{\infty }|\left(b_{1},b_{2},\ldots ,b_{k}\right)=\left(a_{1},a_{2},\ldots ,a_{k}\right)\right\}$$

for $k\in N;a_{1},a_{2},\ldots ,a_{k}\in \left\{0,1,\ldots ,N-1\right\}$ defined by

$$\mu_{p}\left(C_{k}\left(a_{1},a_{2},\ldots ,a_{k}\right)\right)=\prod_{n=1}^{k}p_{a_{n}}.$$

for such cylinder sets. σ is $\mu_{p}$-preserving and ergodic.

**Lemma** **A6.**
*Let σ be a Bernoulli shift on ${\left\{0,1,\ldots ,N-1\right\}}^{\infty }$ for some N∈N and $p=\left(p_{0},p_{1},\ldots ,p_{N-1}\right)\in {\left[0,1\right]}^{N}$ be a stochastic vector. Let $\mu_{p}$ be defined as above and, for k∈N, let*

$$C_{k}=\left\{C_{k}\left(a_{1},a_{2},\ldots ,a_{k}\right)|a_{1},a_{2},\ldots ,a_{k}\in \left\{0,1,\ldots ,N-1\right\}\right\}.$$

*Then,*

$$h\left(\sigma ,C_{k},q\right)=H\left(p,q\right)$$

*holds true for all q≠1, k∈N.*

**Proof.** Take k∈N and q∈R. Notice that

$$C_{k}^{\left(n\right)}=\overset{n-1}{\underset{t=0}{⋁}}\sigma^{-1}\left(C_{k}\right)=C_{k+n}$$

for all n∈N. This implies that

$$\begin{array}{ccc} H(C_{k}^{\left(n\right)}) & = & \frac{-1}{q-1}\log\left(\sum_{P\in C_{k+n}}\mu_{p}{\left(P\right)}^{q}\right)\\ & = & \frac{-1}{q-1}\log\left(\sum_{a_{1},\cdots ,a_{k+n}\in \left\{0,1,\ldots ,N-1\right\}}{\left(\prod_{i=1}^{k+n}p_{i}\right)}^{q}\right)\\ & = & \frac{-1}{q-1}\log\left(\sum_{a_{1},\cdots ,a_{k+n}\in \left\{0,1,\ldots ,N-1\right\}}\left(\prod_{i=1}^{k+n}p_{i}^{q}\right)\right)\\ & = & \frac{-1}{q-1}\log\left({\left(\sum_{a\in \{0,1,\ldots ,N-1\}}p_{a}^{q}\right)}^{k+n}\right)\\ & = & \left(k+n\right)\cdot \frac{-1}{q-1}\log\left(\sum_{a\in \{0,1,\ldots ,N-1\}}p_{a}^{q}\right)\\ & = & \left(k+n)\cdot H(p,q\right) \end{array}$$

for all n∈N and q≠1. Therefore,

$$\begin{array}{ccc} h(\sigma ,C_{k},q) & = & \underset{n\to \infty }{lim inf}\frac{1}{n}H\left(\overset{n-1}{\underset{i=0}{⋁}}\sigma^{-1}\left(C_{k}\right),q\right)\\ & = & \underset{n\to \infty }{lim inf}\frac{k+n}{n}\cdot H\left(p,q\right)=H\left(p,q\right) \end{array}$$

holds true for all q≠1.  □

**Example** **A1.**
*Take any q>1. Choose some N∈N and a stochastic vector $p=\left(p_{0},p_{1},\ldots ,p_{N-1}\right)\in {\left[0,1\right]}^{N}$ such that*

H(p,q)+log2<H(p)

*holds true. This is always possible due to Lemma A5. Further, let $\mu_{p}$ be the probability measure defined before Lemma A6.*

*In order to obtain a map with finitely many monotone parts, identify Bernoulli shift σ on ${\left\{0,1,\ldots ,N-1\right\}}^{\infty }$ with interval map T on Ω=[0,1] defined by*

T(ω)=N·ωmod1

*for all ω∈Ω. This is possible since the correspondence*

$${\left(b_{n}\right)}_{n\in N}\in \left\{0,1,\ldots ,N-1\right\}\mapsto \sum_{n=1}^{\infty }b_{n}N^{-n}$$

*turns σ into T. In this way, measure $\mu_{p}$ can be considered as a measure on *Ω* with the Borel sets. One easily sees that M={[(i−1)/N,i/N]∣i∈{1,2,…,N}} is a partition into intervals on each of which T is monotone and continuous. According to the above identification, M can be considered as the partition $C_{1}$ in Lemma A6. Now*

$$\begin{array}{c} \bar{\mathrm{PE}}\left(T,q\right)\leq h\left(T,M,q\right)+\log2=H\left(p,q\right)+\log2<H\left(p\right)=h\left(T,q\right) \end{array}$$

*holds true. Here the first equality follows from Corollary A1, the last one from Theorem 1, and the first inequality from Lemma A6.*
